# Leaf Movements of Indoor Plants Monitored by Terrestrial LiDAR

**DOI:** 10.3389/fpls.2018.00189

**Published:** 2018-02-16

**Authors:** Mónica Herrero-Huerta, Roderik Lindenbergh, Wolfgang Gard

**Affiliations:** ^1^Department of Geoscience and Remote Sensing, Delft University of Technology, Delft, Netherlands; ^2^TIDOP Research Group, Higher Polytechnic School of Avila, University of Salamanca, Avila, Spain; ^3^Agronomy Department, Purdue University, West Lafayette, IN, United States; ^4^Department of Structural and Building Engineering, Delft University of Technology, Delft, Netherlands

**Keywords:** leaf movements, plants, terrestrial LiDAR, indoor, temporal series

## Abstract

Plant leaf movement is induced by some combination of different external and internal stimuli. Detailed geometric characterization of such movement is expected to improve understanding of these mechanisms. A metric high-quality, non-invasive and innovative sensor system to analyze plant movement is Terrestrial LiDAR (TLiDAR). This technique has an active sensor and is, therefore, independent of light conditions, able to obtain accurate high spatial and temporal resolution point clouds. In this study, a movement parameterization approach of leaf plants based on TLiDAR is introduced. For this purpose, two *Calathea roseopicta* plants were scanned in an indoor environment during 2 full-days, 1 day in natural light conditions and the other in darkness. The methodology to estimate leaf movement is based on segmenting individual leaves using an octree-based 3D-grid and monitoring the changes in their orientation by Principal Component Analysis. Additionally, canopy variations of the plant as a whole were characterized by a convex-hull approach. As a result, 9 leaves in plant 1 and 11 leaves in plant 2 were automatically detected with a global accuracy of 93.57 and 87.34%, respectively, compared to a manual detection. Regarding plant 1, in natural light conditions, the displacement average of the leaves between 7.00 a.m. and 12.30 p.m. was 3.67 cm as estimated using so-called deviation maps. The maximum displacement was 7.92 cm. In addition, the orientation changes of each leaf within a day were analyzed. The maximum variation in the vertical angle was 69.6° from 12.30 to 6.00 p.m. In darkness, the displacements were smaller and showed a different orientation pattern. The canopy volume of plant 1 changed more in the morning (4.42 dm^3^) than in the afternoon (2.57 dm^3^). The results of plant 2 largely confirmed the results of the first plant and were added to check the robustness of the methodology. The results show how to quantify leaf orientation variation and leaf movements along a day at mm accuracy in different light conditions. This confirms the feasibility of the proposed methodology to robustly analyse leaf movements.

## Introduction

Plant canopy structure properties and their spatial changes, are linked to different vegetation processes, such as radiation absorption, plant water balance, precipitation interception and photosynthetic activity (Harley and Baldocchi, 1995). Canopy structural and biochemical variables are frequently used as constraints to model interactions between the land surface and the atmosphere (Sellers et al., 1997). Moreover, many studies that investigated the implications of interception and light transmission for species competition, biodiversity, ecosystem and agro-ecosystem dynamics, as well as wood production, depend on the spatial distribution of leaves and branches (Pretzsch, 2009).

In ecology and plant physiology, circadian rhythms are activities that occur on a near-24-h cycle due to ecologically useful adaptions, regarding plant's physiology and its environment (Sadava et al., 2009). In this context, the growth patterns of roots and leaves are determined by the circadian clock and leaf starch metabolism (Ruts et al., 2012). Therefore, diel leaf movements are a well-typified symbol of the circadian clock (Farré, 2012). These movements have been studied intensively for a long time (Barak et al., 2000). Two reason are notably identified by scientific researches as operators of these fluctuations, plant water balance (Chapin et al., 2011), and photoperiodism (Sysoeva et al., 2010), related to the fluency rate of photosynthetically active radiation in the plant. To optimize the interception of incoming light and to avoid temperature related stress, the leaf angle is adjusting (Medina et al., 1978; Ehleringer, 1998). Plant imaging aims to analyse and quantify the development, growth, physiological and other phenotypic plant properties by different accurate and automated processes. A complete review focuses on the latest advances in high-throughput image-based plant phenotyping (Fahlgren et al., 2015). Although many successes are reported, image based techniques may require the use of a flash during low-light conditions, while some effort is needed in setup and/or processing to manually or automatically obtain 3D results.

TLiDAR has newly arisen as a promising tool to fast measure 3D vegetation structure at plot level with high spatial resolution and millimeter accuracy (Dassot et al., 2010). Light Detection And Ranging (LiDAR) is an active remote sensing technique that accurately measures distances by transmitting laser energy and detecting the time of arrival of the return energy. As an active technique, it is almost insensitive to varying external lighting conditions and is able to capture data even in absence of light. TLiDAR data are extensively used in engineering applications to monitor morphological terrain changes (Herrero-Huerta et al., 2016). The interest on TLiDAR for vegetal plot measurements started in the past decade in forestry (van Leeuwen and Disney, 2018) and (Thies and Spiecker, 2004). Recently, a fully-automatic approach for tree structural modeling at plot level has been proposed in Raumonen et al. (2013). Circadian rhythms in tree geometry can be accurately monitored at millimeter scale and sub-hour temporal resolution by TLiDAR in outdoor conditions (Puttonen et al., 2016). Dornbusch et al. analyse circadian movements and rhythmic leaf growth of Arabidopsis rosettes using a near-infrared laser scanner (Dornbusch et al., 2014). Low cost 3D imaging tools were used for this purpose in Paulus et al. (2014) and evaluated with respect to the metric quality of estimated geometric plant features. To summarize, TLiDAR has potential to monitor vegetation geometry at different scale levels. In addition, scanning can be done outdoors, is insensitive to vary lighting conditions and is able to operate at up to temporal resolutions of minutes. The scanning mechanism is based upon a fast rotating multi-facet polygonal mirror, so little effect is transmitted to plants. As a disadvantage, point clouds cannot provide direct biochemical parameters from plants, as hyperspectral and thermal chlorophyll fluorescence imaging do (Fiorani and Schurr, 2013). On the other hand, image based-3D modeling needs a significant amount of post-processing, while TLiDAR requires a higher cost.

Goal of this research is to show how plant moves can efficiently be captured and parameterized using TLIDAR. In general, data capturing using TLIDAR is considered relatively easy. However, there is still a lack of methodology and corresponding software tool to automatically extract geometric parameters from point cloud data sampling moving plants. Thus, the goal of this study is to propose a phenotyping pipeline to automatically determine movement from leaves in indoor plants through TLiDAR.

The paper is organized as follows: after this brief introduction, the proposed methodology is described in section Methodology. Next, the results and the discussion are presented in section Experimental Results and Discussion, respectively. Finally, conclusions are drawn.

## Methodology

### Experimental setup

**Figure 1:**
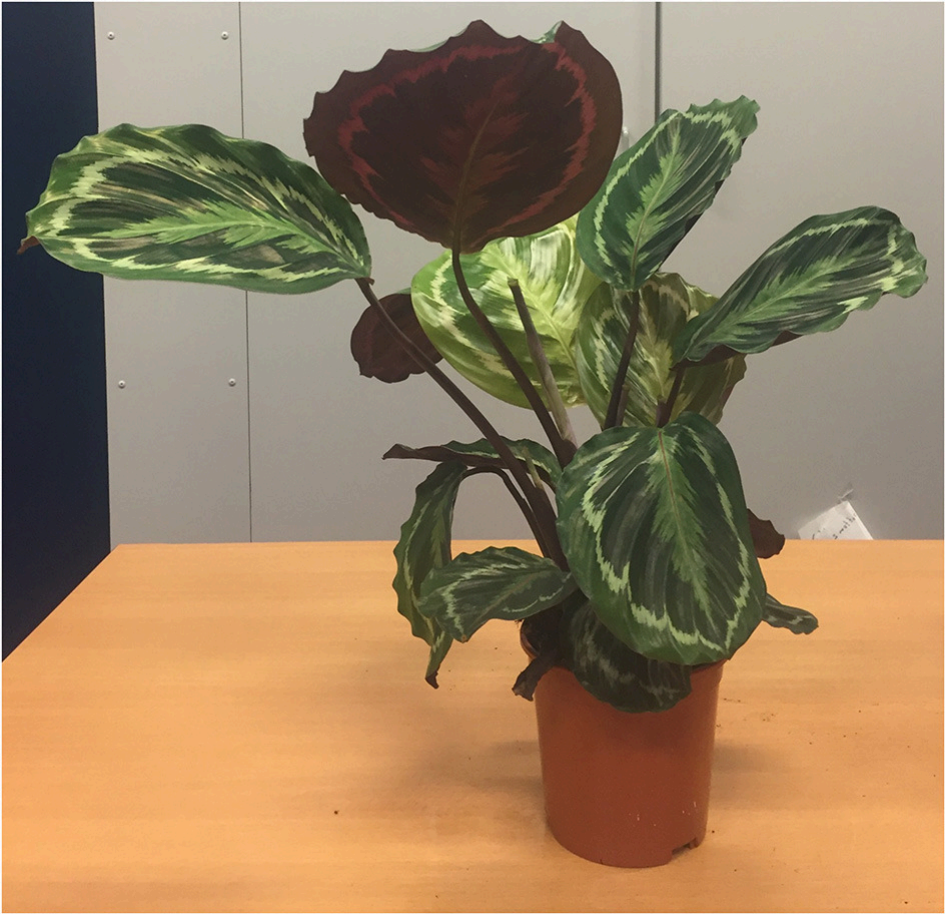
Picture of *Calathea roseopicta* plant 2.

An experiment was carried out with the understory species *Calathea roseopicta* (Linden) Regel, a native species from Tropical America, with basal leaves growing in a rosette shape. Because of the photo- and thermotropism of most tropical plants, *Calathea* unfurl its leaves and moves the whole system including the stem toward the light to optimize the surface for sunlight absorption. During the dark period (night) the leaves coil up and minimize the surface to prevent the plant from cooling-off. Therefore, this species is suitable to monitor the complex, spatial movement of the leaf-stem system. It needs a minimum temperature of 16°C so in temperate systems, it is commonly kept in indoor conditions. Its non-lignified tissues allow the presence of soft tissues susceptible to soil water and light conditions (Chao-ying, 2009). Two plants were tested, planted in an 850 cm^3^ PVC container filled with a substrate composed of sand and a high content of organic material. Studied plant #1 had a mean height of ~45 cm and a canopy diameter of ~31 cm during the measurements, while plant #2 has ~44 cm and ~29 cm, respectively, as Figure 1 illustrates.

The experiment was performed in December 2016. Air temperature (°C) and relative humidity (%) were continuously monitored during the sampling period with a HOBO U30-NRC weather station, with sampling intervals of 5 min. Light conditions were measured during the experiment by the “LuxMeterPro Advanced App” from AM PowerSoftware, with 12 lux-accuracy for a distance <5 m. A Leica Scan-Station C10 scanner, a high speed TLiDAR implementing the so-called time-of-flight principle, is used to collect data (Vosselman and Maas, 2010)^1^. Obstructions in the visibility w.r.t. the position of the laser scanner is hindering complete sampling of the plant surface. Indeed, leaves in the front, as seen from the scanner position, may occlude leaves in the back. Thereby, the relative horizontal position of the TLIDAR w.r.t. the scanned plant should be carefully chosen to have best coverage of leaves within a single scan. Using multiple scans could mitigate this occlusion effect but would (i) include larger time differences within one acquisition and (ii), would require an additional matching or registration step to align the scans as obtained from different positions into a common coordinate system.

Both plants were located in a room, 1.6–1.8 m from the single window oriented in SW direction. Scans were performed at 2 different days. The first sampling day (test A) was done under natural light conditions. Previously to these TLiDAR measurements, the plants were kept in full light conditions. After a 2 day-period in which the plants were kept in complete darkness, the second sampling (test B) was carried out in the dark. An overview of the acquired data is shown in Table 1.

**Table 1:** Overview of the acquired data.

**Test**	**Condition**	**Plant**	**#points in Point Cloud**		
			**7:00 a.m**	**12:30 a.m**	**6:00 p.m**
A	Natural light	1	98,868	94,043	93,235
		2	84,966	82,727	88,597
B	Darkness	1	94,355	96,456	95,765
		2	81,916	82,254	77,008

### Method of leaf movement estimation

#### Outlier removal

The point cloud sample of the plants may contain outliers and noisy points caused by various reasons such as backscatter from interference effects (Thies and Spiecker, 2004). Such points are not regarded as samples of the actual plant and the first step is to filter them from the point cloud. To remove separate isolated points or few-point clusters, a statistical analysis of the distances is performed as follow. First, all points are visited. For each query point, all neighboring points within a pre-set distance are determined and all distances from the query point to these points are determined. After visiting all points, the mean and standard deviation of these distances ($\bar{d}$ and σ_*d*_, respectively) is stored. Next, a threshold (*L*_*d*_) is defined as:

(1) $$\sum =\frac{{\left(X-\bar{X}\right)}^{T}(X-\bar{X})}{\text{k}}=\left(\begin{array}{c} \sigma_{x}^{2}\;\sigma_{xy}\;\sigma_{xz}\\ \sigma_{xy}\;\sigma_{y}^{2}\;\sigma_{yz}\\ \sigma_{xz}\;\sigma_{yz}\;\sigma_{z}^{2} \end{array}\right)$$

where *p* is the confidence level, expressed as the critical value associated in the standard normal density curve (Palnick, 2014).

Again, all points are visited. For a given point, when its average distance to its *k* neighbors is above *L*_*d*_, it is marked as an outlier and removed (Rusu et al., 2008). This method is reasonably fast once the points are organized in a data structure like an octree.

#### Point cloud registration and deviation map calculation

Scans are taken without moving the TLiDAR during the sampling periods. Still, it is not guaranteed that the scans obtained at different moments are fully aligned. Therefore, the registration or alignment of the 3D point clouds is checked using 7 external spherical targets of 10 cm-diameter from Leica©. This registration step estimates a rigid body transformation (only translations and rotations can be applied to the point clouds). The Iterative Closest Point algorithm by Besl and McKay (1992) is used, reaching a negligible mean absolute error among targets from multi-temporal datasets.

After that, all the point clouds are cropped using a common bounding box. At this point, the recognition of correspondences among point clouds is essential to compute plant movements of the plant as displacements between multi-temporal datasets. Therefore, first cloud-to-cloud differences between sequential TLiDAR datasets are computed using a chamfer distance approach that exploits an octree organization (Akmal Butt and Maragos, 1998). Second, the distance statistics resulting from this first step are used to refine the displacements. A more precise cloud-to-cloud distance between two multi-temporal point clouds is extracted by applying a local approximation model to the reference cloud by a quadric surface. As a result, a deviation map of displacements between point clouds is obtained which precisely outlines the coordinates and enables the quantification of plant movements.

#### Segmentation in individual leaves

The changes in the orientation of the leaves along time are estimated from the point cloud data. For that purpose, individual plant leaves are extracted from the point clouds using a segmentation method based on an organization of the point cloud in an octree structure, as proposed in Woo et al. (2002).

Initially, the point normals are estimated by Delaunay triangulation (Golias and Dutton, 1997). Next, the 3D points are organized in an octree structure, where the local point normals are used as a criterion for splitting an octree cell: if points at a certain octree depth all have similar normals, subdivision terminates; otherwise, the octree cell is divided. This process is repeated until normals stabilize at a certain depth or the maximal octree depth is reached. After splitting is finished, adjacent cells having similar orientation are merged in a bottom-up procedure. First, cells with a large variation in normals are simply removed. Such behavior might, for example, occur at the edges of leaves. Next, an optimally flat cell is selected as a seed cell and adjacent cell are added as long as a homogeneity criterion is met. When such segment is finished, a new seed is selected and the procedure is repeated using the remaining cells. The standard deviation of the normal vectors in a cell is taken as homogeneity criterion. After termination of this procedure, the point cloud is automatically separated into certain regions by considering the obtained segments. Using a suitable criterion value, it is possible to tune the method such that the resulting segments correspond to individual leaves.

The last step is to match individual leaves, which is difficult as the individual leaves are moving and changing the shape between the different data acquisitions. Automatic co-registration of each leaf point cloud segment is done by least squares matching of overlapping surfaces (Least Squares 3D Surface Matching). The transformation parameters of the 3D compared surface (leaf segment after movement) to a 3D reference surface (leaf segment before movement) is approximated by a so-called Generalized Gauss–Markov model, based on minimizing the squared sum of Euclidean distances between surfaces (Gruen and Akca, 2008).

#### Leaf normal calculation

Once the leaves are segmented, leaf-wise orientation is approximated by the leaf normal. For this purpose, Principal Component Analysis (PCA) is used (Weinmann et al., 2014). This statistical analysis uses the first and second moments of the points and results in three orthogonal vectors centered on the center of gravity of the point cloud. The PCA synthesizes the distribution of points along the three dimensions and therefore models the principal directions and magnitudes of variation of the point distribution around the center of gravity.

The coordinates *x*_*i*_, *y*_*i*_, and *z*_*i*_ for each point *i* = *1,… k* from the point cloud of each leaf, is considered. The covariance matrix (Σ) (2) of each leaf point cloud (*X*) is defined by:

(2) $$\theta =\text{arctan}\frac{\text{y}}{\text{x}}\;$$

where ($\sigma_{x}^{2}$, $\sigma_{y}^{2}$, $\sigma_{z}^{2}$) are the variances of the coordinate directions and the elements outside the main diagonal of Σ are the covariances. $\bar{X}$ contains *k* copies of the mean of the three coordinates of the leaf point cloud (*X*). The principal leaf normal is the third eigenvector of Σ (Jolliffe, 2002).

#### Computation of orientation variation

The orientation variation of each leaf is characterized in a spherical coordinate system by determining the change in both spherical azimuth (θ) (aspect) and elevation angle (Φ) (hyponasty). For this reason, firstly, a subtraction of the normals from each leaf at different times is done, always in temporal order with respect to the first epoch. These angles obtained from the difference-in-normal vector *(x, y, z)* are calculated as follows:

(3) $$\emptyset =\text{arctan}\frac{\text{z}}{\sqrt{{\text{x}}^{\text{2}}{+\text{y}}^{\text{2}}}}$$

**Figure 2:**
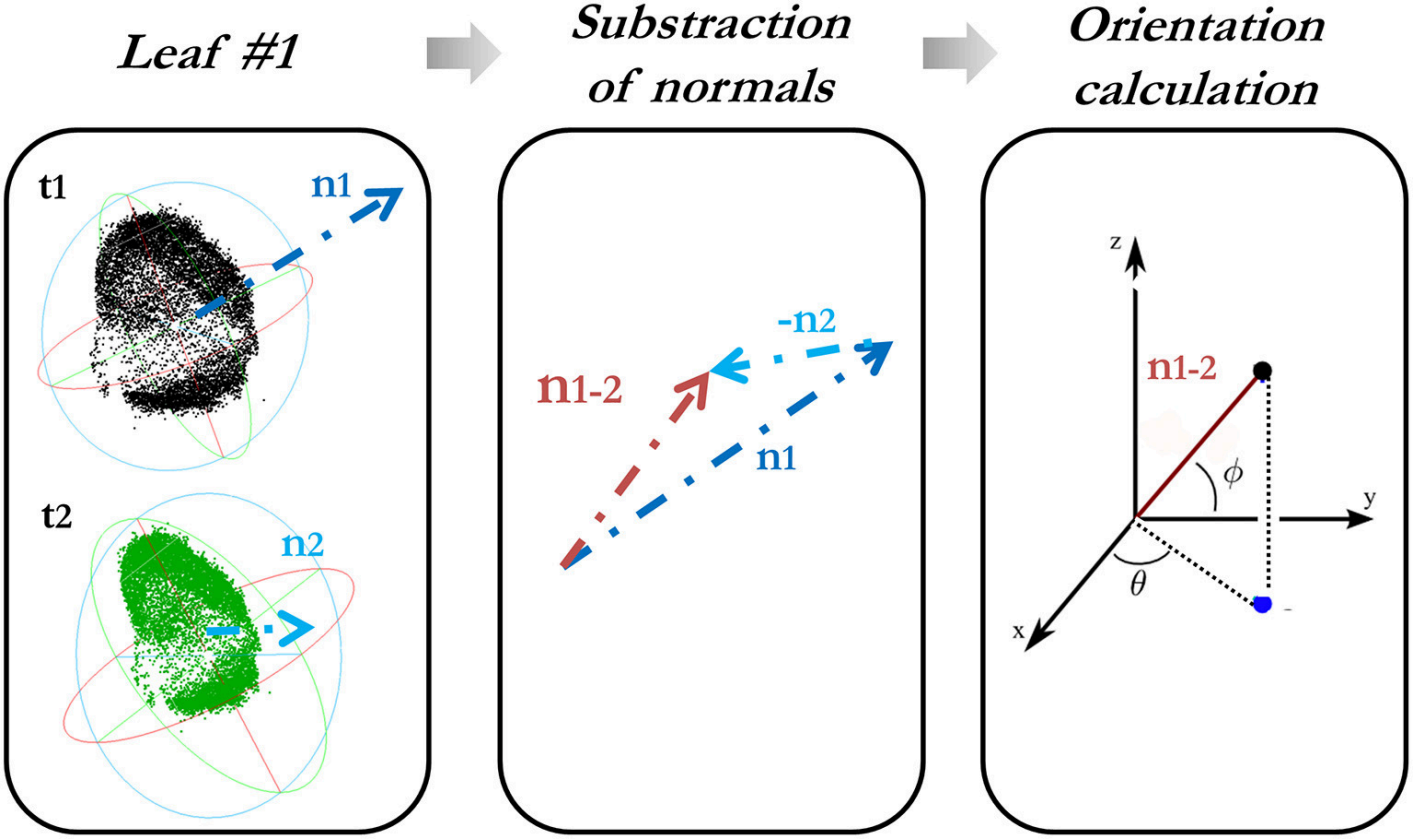
Calculation of the orientation variation of each leaf along the time.

Figure 2 explains how the computation of the orientation variation is carried out.

#### Volume variations by canopy mesh

The foliage shape of the full plant may help to understand the radiation regime and canopy structure. However, the single scan acquisitions only give a partial point cloud due to the one-sided field of view of the TLiDAR. This paragraph explains how to quantify the canopy volume variation from the point cloud of the plant.

The approach is supported by a 3D modeling of the plant using a 3D Delaunay triangulation (Golias and Dutton, 1997), as used before for the leaf segmentation (section Segmentation in Individual Leaves). The result is a 3D mesh. An additional filter phase is required to obtain a plant canopy model close to reality. The approximation shown in Attene (2008) is used in this step which incorporates several smoothing operators to repair issues in the mesh like holes and different types of noise (Fan et al., 2008).

Once the canopy mesh is fixed, its shape is approximated by a 3D convex hull (Barber et al., 1996). The convex-hull is chosen due to its capacity of enveloping the effective area which potentially can be related to the radiance absorption. As an alternative in future, an alpha-shape approximation of the hull could be used, which is able to more flexible estimate the shape of the plant canopy, by varying the alpha coefficient.

As single scans only provide partial point clouds, the recovery of the less well-visible backside is achieved through symmetry. Therefore, the symmetry axis is estimated by a 180° field of view from the dataset. Finally, the canopy shape is obtained which allows the direct volume estimation and changes along time.

## Experimental results and discussion

Sunrise and sunset times were similar during the experiment. Therefore, the sampling periods are comparable. The plant substrate was maintained well-watered to reduce the effect of root water shortage. The room temperature remained constant during the sampling period; however the relative humidity changed between test A and B (Table 2). This variation in humidity could have led to stress or more water movement within the plants in test B.

**Table 2:** Room conditions during test A and B [sunrise and sunset times provided in GMT(+1)].

**Test**	**Date**	**Timing**		**Variables**	
		**Sunrise**	**Sunset**	**Temp**.	**Rel. humidity**
A	02/12/2016	8:30 a.m	16:34 p.m	21.2°	47.4 %
B	05/12/2016	8:34 a.m	16:32 p.m	21.2°	32.2 %

**Figure 3:**
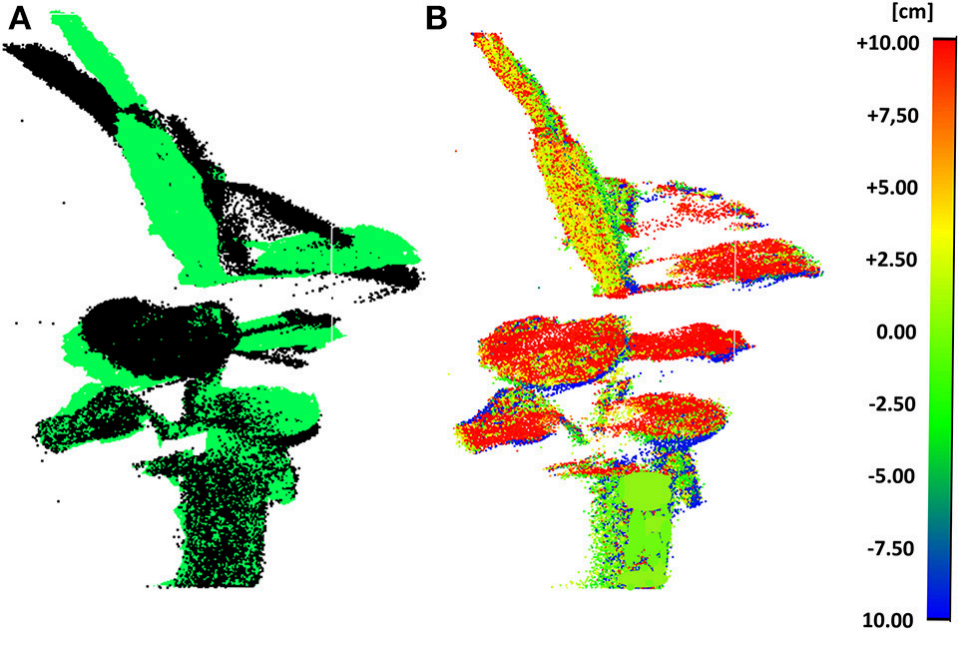
Scans at 7.00 a.m. and at 12.30 p.m. of plant 1 during test A: superimposed (7.00 a.m. in black color and 12.30 p.m. in green color) **(A)** and deviation map **(B)**.

The scan pre-processing settings to remove noise (section Outlier Removal) from the data were fixed to a neighborhood size of a 1.6 cm sphere diameter. The leaf movements of *C. roseopicta* plant 1 are illustrated in Figure 3, by superimposing the scan obtained at 7.00 a.m. to the one obtained at 12.30 p.m. during test A (Figure 3A) and by calculating the deviation map from the compared point cloud (at 12.30 p.m.) to the reference point cloud (at 7.00 a.m.) (Figure 3B), as section Point Cloud Registration and Deviation Map Calculation explains. This time interval was chosen as during this time the biggest movement in terms of leaf displacement occurred. Further estimations can be obtained from this product. For instance, the maximum displacement of 7.92 cm between the two scans occurs at leaf #7. The average distance movement of this leaf is 3.67 cm.

**Figure 4:**
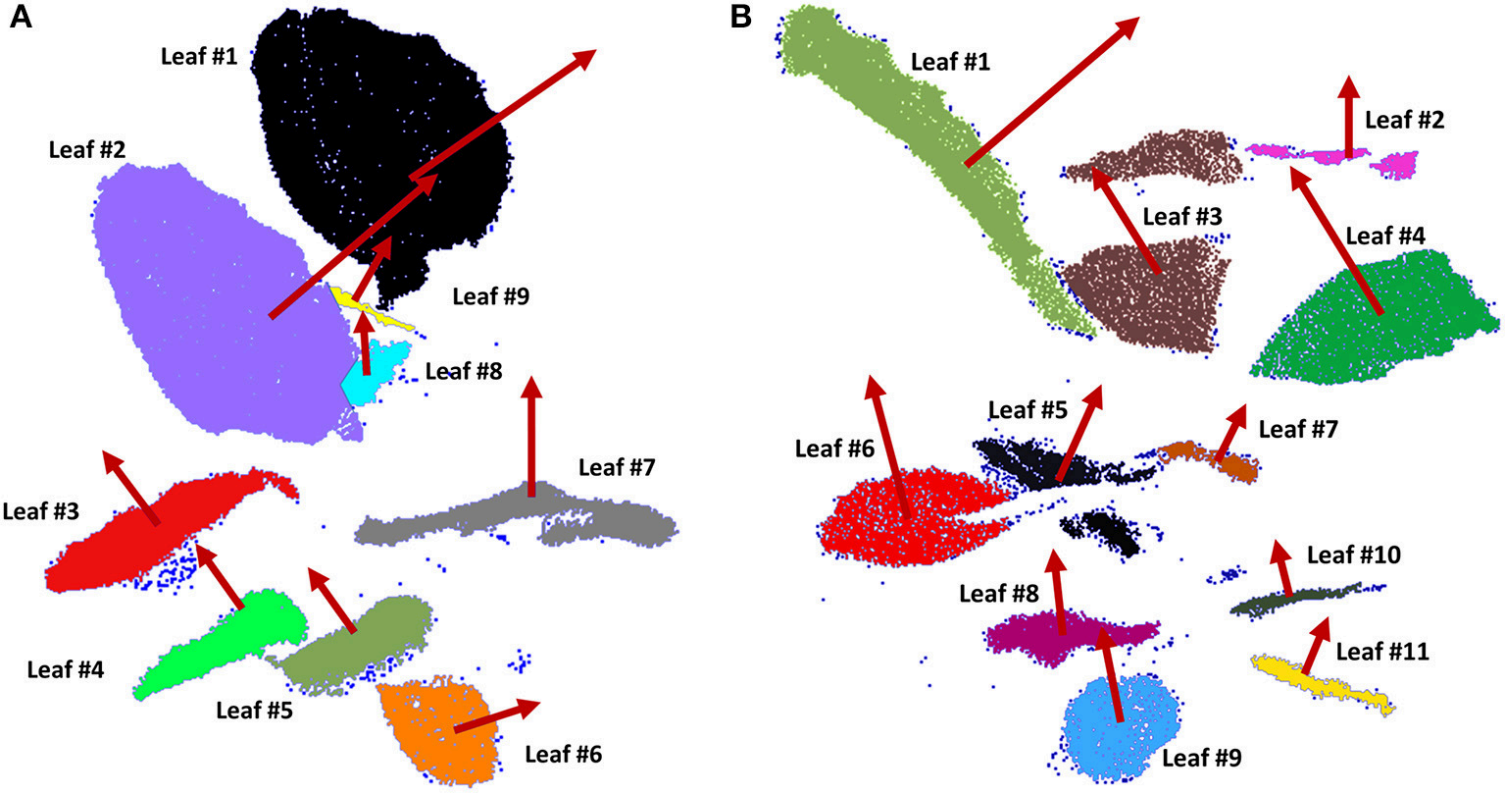
Individual leaves of plant 1 **(A)** and plant 2 **(B)** extracted using an octree-based segmentation (leaves in different colors) and consecutive orientation estimation by PCA (normal vector in red of each leaf).

In order to apply the segmentation method to extract individual leaves, some parameters have to be fixed; notably, the minimum voxel size and the number of iterations. These parameters were set as 1.8 cm and 5 iterations, respectively. Furthermore, the standard deviation of point normals within a cell as a tolerance for subsequent subdivisions, was set at 6 cm. To perform the Least Square Matching for individually matching the leaves among multi-temporal datasets, the iteration criteria threshold was set as 3.8 cm. As a result, 9 leaves of plant 1 and 11 leaves of plant 2 were recognized from all the scans. Figure 4 shows the individual leaves extracted from both plants at 7.00 a.m. (test A) in different colors, where normals in red are obtained by PCA. Outliers are colored in dark blue.

**Figure 5:**
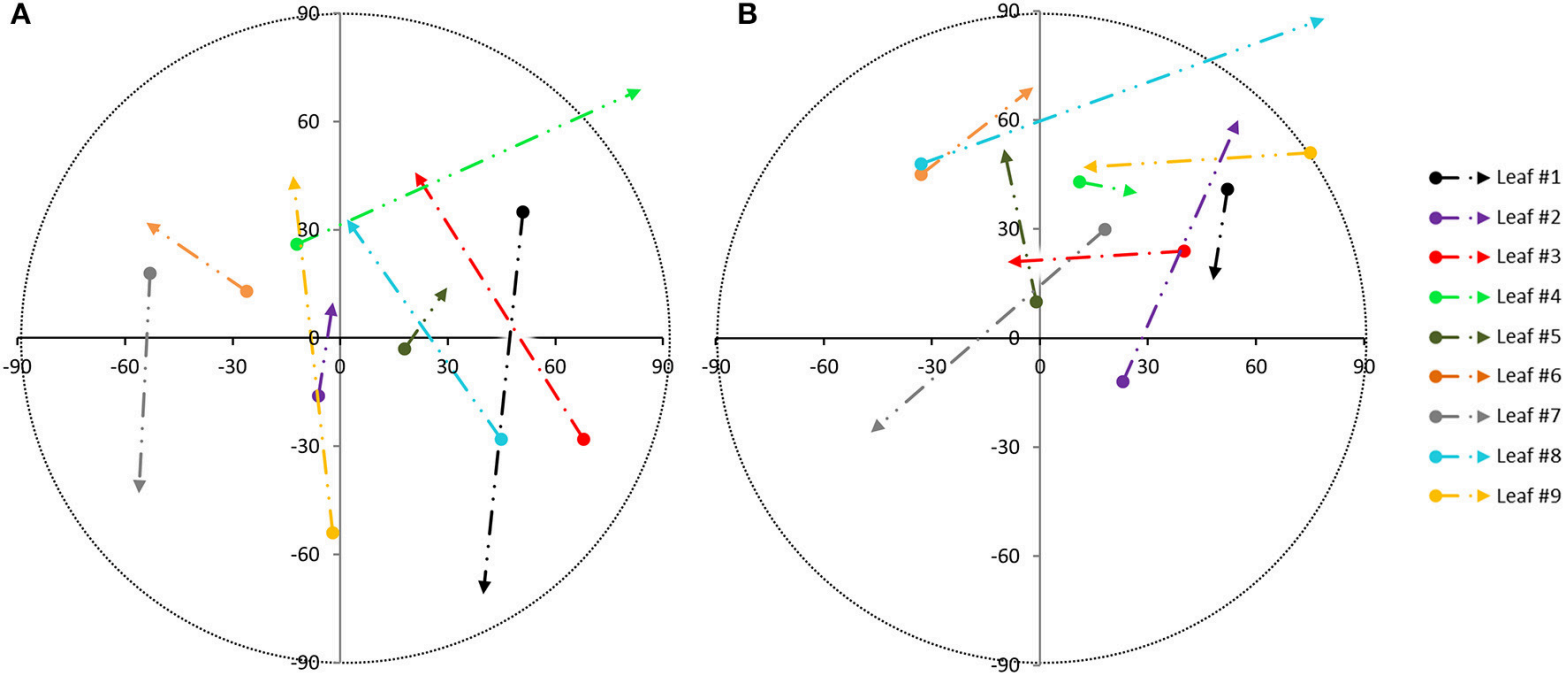
Movement pattern from individual leaves of plant 1, based on the angle orientation change within sampling periods: during test A **(A)** and during test B **(B)** [azimuth changes on x axis and vertical angle changes on y axis from 7.00 a.m. to 12.30 p.m. (beginning of the arrow) and from 12.30 p.m. to 6.00 p.m. (end of the arrow)].

The global accuracy reached was analyzed by comparison to a manual segmentation of the leaves. To do so, each leaf is considered a target class and for each point. It can be determined if it is classified in the correct class. Results are collected in a confusion matrix (not shown), (Sasidharan et al., 2010). The resulting global accuracy was found to be 93.57% for plant 1 and 87.34% for plant 2. The plant species morphology gives the opportunity to use the proposed automatic segmentation technique due to the fact that the leaves and stems have dissimilar orientations. Once the individual leaves are obtained, the variability of each leaf orientation along the sampling tests is estimated. Figure 5 shows the variation in orientation from the leaves of plant 1 during test A (Figure 5A) and during test B (Figure 5B). These variations were first derived between 7.00 a.m. and 12.30 p.m. (beginning of the arrow) and next between 12.30 and 6.00 p.m. (end of the arrow). These intervals were chosen because during these intervals, movement was bigger. The azimuth changes are represented on the *x*-axis while the vertical angle change is on the *y*-axis (both in sexagesimal degrees), taking into account the normal direction of each leaf as obtained by PCA. The color of each trajectory corresponds to the leave color in Figure 1. Different light conditions clearly correspond to different movement directions. The orientation change in darkness (Figure 5B) is smaller than during natural light conditions (Figure 5A).

The results of the orientation variations from plant 2 are displayed in Table 3. A similar magnitude order in the orientation variation is reached regarding plant 1 during both tests.

**Table 3:** Results of orientation variation from plant 2 during test A and B.

**Test**	**#Leaf**	**Azimuth changes**		**Vertical angle changes**	
		**7.00–12.30**	**12.30–18.00**	**7.00–12.30**	**12.30–18.00**
A	#1	−58°	−54°	−56°	−64°
	#2	47°	−22°	51°	27°
	#3	20°	69°	−3°	−53°
	#4	−7°	15°	−20°	−24°
	#5	−42°	18°	45°	−14°
	#6	−83°	24°	−88°	−21°
	#7	−11°	−72°	−32°	−10°
	#8	−55°	81°	−70°	−64°
	#9	31°	31°	34°	−55°
	#10	13°	−13°	−45°	−39°
	#11	−7°	−2°	60°	−47°
B	#1	−63°	75°	75°	−71°
	#2	19°	−18°	35°	49°
	#3	−70°	7°	85°	−45°
	#4	52°	37°	34°	−52°
	#5	−5°	−18°	5°	−28°
	#6	9°	47°	9°	58°
	#7	−70°	−79°	−50°	83°
	#8	6°	81°	46°	61°
	#9	18°	−23°	−39°	12°
	#10	79°	28°	−88°	1°
	#11	0°	18°	63°	36°

From the scan analysis results, we conclude that *C. roseopicta* displayed significant movement in the leaves during light and dark periods, with different patterns for both tests and plants. This species shows conspicuous movement between the tested light conditions, however this leaf movement can be influenced by growing patterns of young leaves (Puttonen et al., 2016).

Leaf #1 from plant 1, for example, moves in the same direction during both light conditions, but its movement is larger in natural light conditions. In both situations, the starting point is similar: it means that the angle variation between 7.00 a.m. and 12.30 p.m. is comparable. Conversely, the end point is really different (from 12.30 to 6.00 p.m.). A possible explanation is that the movement of this leaf during the afternoon is related to the photosynthesis process, so the leaf movement has less influence for the sun-light in the morning. It can probably be related to the different transpiration rates produced by leaf #1 within a day.

**Figure 6:**
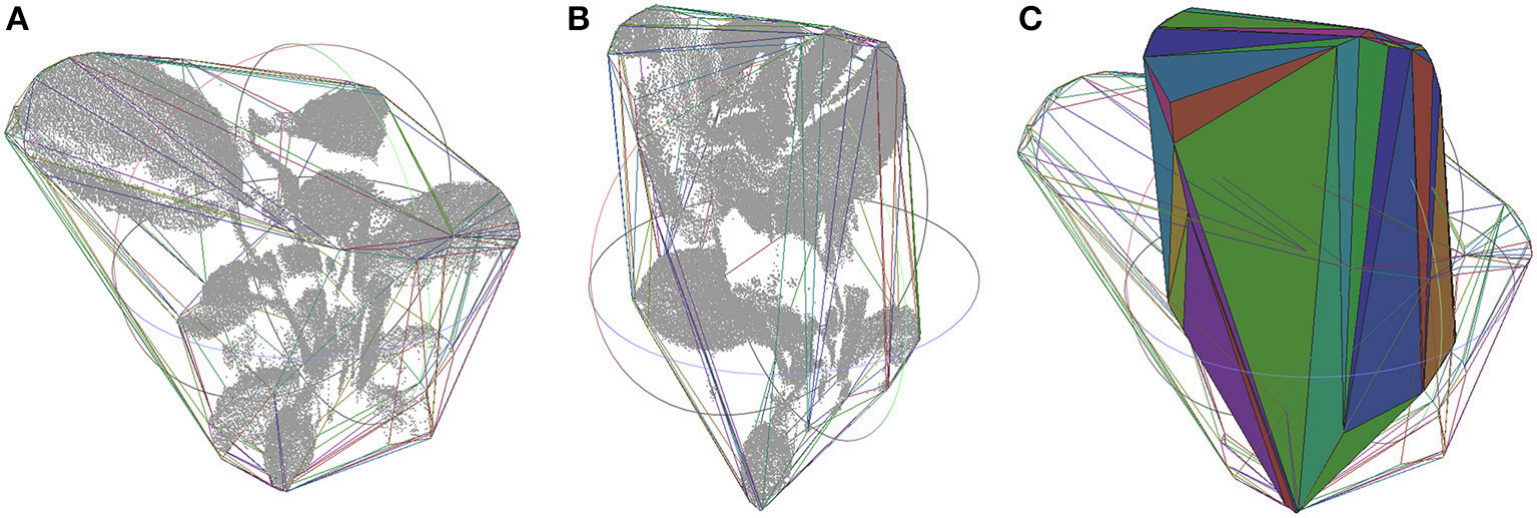
Canopy point cloud at 12.30 p.m. **(A)** and at 6.00 p.m. **(B)** during test A from plant 1, together with its convex hull plotted by lines in random colors depending on the face they belong to. Both convex hulls superimposed, one in lines and the other in solid **(C)**.

Canopy variations along time can actually be quantified. As section Volume Variations by Canopy Mesh describes, the convex-hull of the entire plant is generated from the point cloud. This process is illustrated in Figure 6, where the shape variation of the plant 2 during test A at 12.30 p.m. (Figure 6A) and 6.00 p.m. (Figure 6B) can be clearly defined as ‘closing the leaves’ (Figure 6C). The convex-hull volume was analyzed to extract the variations at different times. Table 4 characterizes these values during the morning and afternoon in test A and B from the two studied plants, proving that in natural light conditions the variation is always bigger.

**Table 4:** Canopy volume analysis by convex-hull.

**Test**	**#Plant**	**Canopy volume (dm**^**3**^**)**			**Volume variation (dm**^**3**^**)**	
		**7:00**	**12:30**	**18:00**	**7:00–12:30**	**12:30–18:00**
A	1	33.12	28.70	31.27	−4.42	2.57
B	1	30.6	30.49	28.96	−0.16	−1.53
A	2	37.24	41.98	25.00	4.74	−16.98
B	2	45.52	43.36	44.80	−2.16	1.44

Thus, our data shows that light conditions influence diel leaf movement, characterized by movement patterns and leaf movement rate. As a general idea, we saw that leaves were opening during the day and closing at night (changing the elevation angle along sampling periods), generating an up and down movement as previous studies already indicated (Manel et al., 2001).

Notice that leaves can generate shadows within plants, occluding certain parts, therefore the obtained 3D point cloud of the plant surface, usually is incomplete. To overcome this challenge, the individual leaf architecture could be reconstructed via a model, already proposed for cereal leaves (Dornbusch et al., 2012).

The biological significance of diel leaf movements is related to favorable conditions of energetic requirements, water availability and auxin responsiveness (Stitt and Zeeman, 2012). This proposition agrees with the maximal peak of movement and orientation variation during the early morning. Moreover, the leaf vertical angle tracks the usual daily temperature oscillations with a top in the late-afternoon. Bridge et al. (Bridge et al., 2013) determined that moving up the leaves is favorable to cool them during the warm daily hours and reduces the radiation contents when it exceeds the photosynthetic capacity.

## Conclusions

This paper shows how TLiDAR can be used to efficiently and non-invasively parameterize plant movement. It presents a phenotyping method to monitor leaf movements from plants by TLiDAR, even in absence of light. We show that this approach is robust and computationally easy to determine, providing a tool to accurately measure complex leaf-stem system movements from plants with high temporal and spatial resolution.

*Calathea roseopicta* species was studied, showing different leaf movements in presence of natural daylight conditions and in darkness. Moreover, canopy variations in volume were analyzed by a convex hull approach. In darkness, the displacements are smaller and with a dissimilar orientation pattern than in natural light conditions. The natural movements are expressed by opening of the leaves during the day and closing them at night, pointing to the source of natural light (the window, in our study case). In absence of light, the plant still showed displacements, but with different pattern and magnitude.

The low relative humidity conditions during both sampling periods may have resulted in an increment in transpiration rates, leading to possible leaf movement during the photosynthesis. As further consideration, water availability should be controlled and sunlight received by the plant should be equal in all directions to rigorously compare multi-temporal datasets.

Using TLiDAR technology to derive vegetation movements open up fresh challenges which merit more methodological advance. The proposed methodology could help to support biological investigations of exchange of water, sunlight absorption and other bio-geochemical between plants and the atmosphere. Furthermore, these achievements would break through the barrier of knowledge in objectively determining the behavior of plants and even its structure and function in ecosystem, nowadays remaining poorly understood (Whippo and Hangarter, 2009). Furthermore, the behavior of interior plants is linked to indoor air quality and this connection will be explored as further studies.

## Author contributions

MH-H designed the experiment, created the software, ran the data analysis and analyzed the data; MH-H and RL wrote the paper; WG edited the paper. All authors read and approved the final manuscript.

### Conflict of interest statement

The authors declare that the research was conducted in the absence of any commercial or financial relationships that could be construed as a potential conflict of interest.

## Footnotes

^1^Available online at: https://hds.leica-geosystems.com (Accessed January 11, 2018).
